# *N*-(2,4,6-Trimethyl­phen­yl)succinamic acid

**DOI:** 10.1107/S1600536809029791

**Published:** 2009-07-31

**Authors:** B. Thimme Gowda, Sabine Foro, B. S. Saraswathi, Hartmut Fuess

**Affiliations:** a Department of Chemistry, Mangalore University, Mangalagangotri 574 199, Mangalore, India; b Institute of Materials Science, Darmstadt University of Technology, Petersenstrasse 23, D-64287 Darmstadt, Germany

## Abstract

The amide bond in the title compound {systematic name: 3-[(2,4,6-trimethyl­phen­yl)amino­carbon­yl]propionic acid}, C_13_H_17_NO_3_, has a *trans* conformation. In the crystal, two mol­ecules form a centrosymmetric dimer connected by pairs of O—H⋯O hydrogen bonds. Inter­molecular N—H⋯O hydrogen bonds link the dimers into a three dimensional network.

## Related literature

For related structures, see: Gowda *et al.* (2009**a* ▶,*b* ▶,c* ▶); Jagannathan *et al.* (1994 ▶). For the modes of inter­linking carboxylic acids by hydrogen bonds, see: Leiserowitz (1976 ▶).
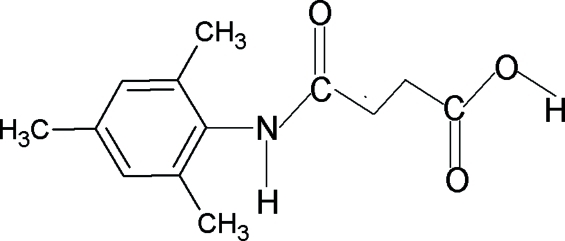


## Experimental

### 

#### Crystal data

C_13_H_17_NO_3_
*M* *_r_* = 235.28
Triclinic, 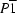

*a* = 4.7646 (4) Å
*b* = 10.859 (1) Å
*c* = 13.111 (2) Å
α = 70.217 (8)°
β = 86.158 (8)°
γ = 79.351 (8)°
*V* = 627.31 (12) Å^3^
*Z* = 2
Cu *K*α radiation
μ = 0.72 mm^−1^
*T* = 299 K
0.55 × 0.25 × 0.08 mm

#### Data collection

Enraf–Nonius CAD-4 diffractometer
Absorption correction: ψ scan (North *et al.*, 1968 ▶) *T*_min_ = 0.692, *T*_max_ = 0.945
3109 measured reflections
2234 independent reflections
1918 reflections with *I* > 2σ(*I*)
*R*_int_ = 0.014
3 standard reflections frequency: 120 min intensity decay: 1.0%

#### Refinement

*R*[*F* ^2^ > 2σ(*F* ^2^)] = 0.048
*wR*(*F* ^2^) = 0.145
*S* = 1.05
2234 reflections
182 parameters
H atoms treated by a mixture of independent and constrained refinement
Δρ_max_ = 0.30 e Å^−3^
Δρ_min_ = −0.30 e Å^−3^

### 

Data collection: *CAD-4-PC* (Enraf–Nonius, 1996 ▶); cell refinement: *CAD-4-PC*; data reduction: *REDU4* (Stoe & Cie, 1987 ▶); program(s) used to solve structure: *SHELXS97* (Sheldrick, 2008 ▶); program(s) used to refine structure: *SHELXL97* (Sheldrick, 2008 ▶); molecular graphics: *PLATON* (Spek, 2009 ▶); software used to prepare material for publication: *SHELXL97*.

## Supplementary Material

Crystal structure: contains datablocks I, global. DOI: 10.1107/S1600536809029791/bt5016sup1.cif

Structure factors: contains datablocks I. DOI: 10.1107/S1600536809029791/bt5016Isup2.hkl

Additional supplementary materials:  crystallographic information; 3D view; checkCIF report

## Figures and Tables

**Table 1:** Hydrogen-bond geometry (Å, °)

*D*—H⋯*A*	*D*—H	H⋯*A*	*D*⋯*A*	*D*—H⋯*A*
N1—H1*N*⋯O1^i^	0.86 (2)	2.10 (2)	2.9368 (18)	163.6 (19)
O2—H2*O*⋯O3^ii^	0.88 (3)	1.80 (3)	2.679 (2)	172 (3)

Symmetry codes: (i) 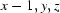; (ii) 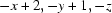.

**Fig. 1.**
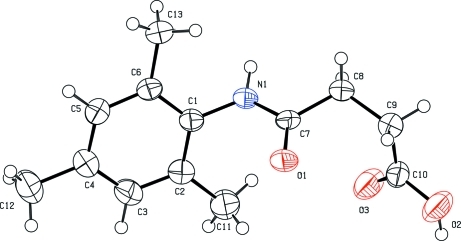
Molecular structure of (I), showing the atom labelling and the displacement ellipsoids are at the 50% probability level. The H atoms are represented as small spheres of arbitrary radii.

**Fig. 2.**
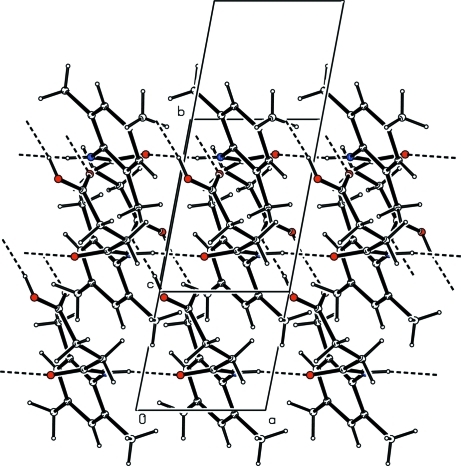
Molecular packing of (I) with hydrogen bonds shown as dashed lines.

## supplementary crystallographic information

### Comment

The amide moiety is an important constituent of many biologically significant compounds. As a part of studying the effect of ring and side chain substitutions on the structures of this class of compounds (Gowda *et al.*, 2009*a,b,c*), the crystal structure of *N*-(2,4,6-trimethylphenyl)-succinamic acid (I) {systematic name: 3-[(2,4,6-trimethylphenyl)-aminocarbonyl]propionic acid} has been determined. The conformations of N—H and C=O bonds in the amide segment of the structure are *trans* to each other (Fig.1). But the conformations of the amide O atom and the carbonyl O atom of the acid segment are *cis* to each other Further, the conformations of the C(O)—C bonds in the N—CO—CH_2_—CH_2_—C(O)—OH segment have "*trans*" and "*gauche*" torsions with the adjacent C—H bonds.

The C=O and O—H bonds of the acid group are in *syn* position to each other, similar to that observed in the crystal structures of *N*-(2,6-dimethylphenyl)- succinamic acid (Gowda *et al.*, 2009*b*) and *N*-(2-chlorophenyl)succinamic acid (Gowda *et al.*, 2009*a*) but in contrast to the *anti* positions observed in the structure of *N*-(3,5-dichlorophenyl)succinamic acid(Gowda *et al.*, 2009*c*)

The torsional angles of the groups, C1 - N1 - C7 - C8, N1 - C7 - C8 - C9, C7 - C8 - C9 - C10 and C8 - C9 - C10 - O2 in the side chain are 180.0 (2)°, -160.5 (2)°, 63.5 (2)° and -174.9 (2)°, respectively. The N—H···O and O—H···O intermolecular hydrogen bonds pack the molecules in the structure into supramolecular chains (Table 1, Fig.2).

The modes of interlinking carboxylic acids by hydrogen bonds is described elsewhere (Leiserowitz, 1976). The packing of molecules involving dimeric hydrogen bonded association of each carboxyl group with a centrosymmetrically related neighbor has also been observed (Jagannathan *et al.*, 1994).

### Experimental

The solution of succinic anhydride (0.025 mole) in toluene (25 cc) was treated dropwise with the solution of 2,4,6-trimethylaniline(0.025 mole) also in toluene (20 cc) with constant stirring. The resulting mixture was stirred for about one h and set aside for an additional hour at room temperature for the completion of reaction. The mixture was then treated with dilute hydrochloric acid to remove the unreacted 2,4,6-trimethylaniline. The resultant solid *N*-(2,4,6-trimethylphenyl)-succinamic acid was filtered under suction and washed thoroughly with water to remove the unreacted succinic anhydride and succinic acid. It was recrystallized to constant melting point from ethanol. The purity of the compound was checked by elemental analysis and characterized by its infrared spectra. The plate like colourless single crystals of the compound used in X-ray diffraction studies were grown in ethanolic solution by slow evaporation at room temperature.

### Refinement

H atoms bonded to C  were positioned with idealized geometry using a riding model [C—H = 0.93 Å to 0.97Å] with   *U*(H) set to 1.2*U*_eq_(C). The other H atoms were located in a difference map and their position refined with   *U*(H) set to 1.2*U*_eq_(N,O).

### Crystal data

**Table:**

C_13_H_17_NO_3_	*Z* = 2
*M**_r_* = 235.28	*F*(000) = 252
Triclinic, *P*1	*D*_x_ = 1.246 Mg m^−^^3^
Hall symbol: -P 1	Cu *K*α radiation, λ = 1.54180 Å
*a* = 4.7646 (4) Å	Cell parameters from 25 reflections
*b* = 10.859 (1) Å	θ = 4.4–22.9°
*c* = 13.111 (2) Å	µ = 0.72 mm^−^^1^
α = 70.217 (8)°	*T* = 299 K
β = 86.158 (8)°	Plate, colourless
γ = 79.351 (8)°	0.55 × 0.25 × 0.08 mm
*V* = 627.31 (12) Å^3^	

### Data collection

**Table:**

Enraf–Nonius CAD-4 diffractometer	1918 reflections with *I* > 2σ(*I*)
Radiation source: fine-focus sealed tube	*R*_int_ = 0.014
graphite	θ_max_ = 67.0°, θ_min_ = 3.6°
ω/2θ scans	*h* = −5→2
Absorption correction: ψ scan (North *et al.*, 1968)	*k* = −12→12
*T*_min_ = 0.692, *T*_max_ = 0.945	*l* = −15→15
3109 measured reflections	3 standard reflections every 120 min
2234 independent reflections	intensity decay: 1.0%

### Refinement

**Table:**

Refinement on *F*^2^	Secondary atom site location: difference Fourier map
Least-squares matrix: full	Hydrogen site location: inferred from neighbouring sites
*R*[*F*^2^ > 2σ(*F*^2^)] = 0.048	H atoms treated by a mixture of independent and constrained refinement
*wR*(*F*^2^) = 0.145	*w* = 1/[σ^2^(*F*_o_^2^) + (0.0856*P*)^2^ + 0.1672*P*] where *P* = (*F*_o_^2^ + 2*F*_c_^2^)/3
*S* = 1.05	(Δ/σ)_max_ = 0.004
2234 reflections	Δρ_max_ = 0.30 e Å^−^^3^
182 parameters	Δρ_min_ = −0.30 e Å^−^^3^
0 restraints	Extinction correction: *SHELXL97* (Sheldrick, 2008), Fc^*^=kFc[1+0.001xFc^2^λ^3^/sin(2θ)]^-1/4^
Primary atom site location: structure-invariant direct methods	Extinction coefficient: 0.016 (3)

### Special details

**Table:**

Geometry. All e.s.d.'s (except the e.s.d. in the dihedral angle between two l.s. planes) are estimated using the full covariance matrix. The cell e.s.d.'s are taken into account individually in the estimation of e.s.d.'s in distances, angles and torsion angles; correlations between e.s.d.'s in cell parameters are only used when they are defined by crystal symmetry. An approximate (isotropic) treatment of cell e.s.d.'s is used for estimating e.s.d.'s involving l.s. planes.
Refinement. Refinement of *F*^2^ against ALL reflections. The weighted *R*-factor *wR* and goodness of fit *S* are based on *F*^2^, conventional *R*-factors *R* are based on *F*, with *F* set to zero for negative *F*^2^. The threshold expression of *F*^2^ > σ(*F*^2^) is used only for calculating *R*-factors(gt) *etc*. and is not relevant to the choice of reflections for refinement. *R*-factors based on *F*^2^ are statistically about twice as large as those based on *F*, and *R*- factors based on ALL data will be even larger.

### Fractional atomic coordinates and isotropic or equivalent isotropic displacement parameters (Å^2^)

**Table:**

	*x*	*y*	*z*	*U*_iso_*/*U*_eq_	
C1	0.3669 (3)	0.77823 (17)	0.30593 (13)	0.0376 (4)	
C2	0.5805 (4)	0.67491 (18)	0.36121 (15)	0.0452 (4)	
C3	0.6582 (5)	0.6700 (2)	0.46280 (16)	0.0549 (5)	
H3	0.804 (5)	0.598 (2)	0.5015 (19)	0.066*	
C4	0.5306 (4)	0.7610 (2)	0.51124 (15)	0.0512 (5)	
C5	0.3135 (4)	0.8597 (2)	0.45520 (15)	0.0469 (5)	
H5	0.225 (5)	0.923 (2)	0.4870 (18)	0.056*	
C6	0.2278 (3)	0.87030 (18)	0.35308 (14)	0.0400 (4)	
C7	0.4594 (3)	0.81334 (16)	0.11383 (13)	0.0368 (4)	
C8	0.3304 (4)	0.8203 (2)	0.00956 (15)	0.0494 (5)	
H8A	0.214 (5)	0.912 (2)	−0.0210 (18)	0.059*	
H8B	0.205 (5)	0.749 (2)	0.0314 (18)	0.059*	
C9	0.5540 (4)	0.8015 (2)	−0.07431 (16)	0.0543 (5)	
H9A	0.683 (5)	0.867 (2)	−0.0887 (19)	0.065*	
H9B	0.474 (5)	0.815 (2)	−0.142 (2)	0.065*	
C10	0.7408 (4)	0.6682 (2)	−0.04148 (15)	0.0479 (5)	
C11	0.7206 (5)	0.5687 (2)	0.31581 (18)	0.0607 (6)	
H11A	0.8884	0.5946	0.2757	0.073*	
H11B	0.5898	0.5570	0.2686	0.073*	
H11C	0.7730	0.4868	0.3741	0.073*	
C12	0.6208 (6)	0.7531 (3)	0.62154 (18)	0.0727 (7)	
H12A	0.8203	0.7163	0.6316	0.087*	
H12B	0.5120	0.6975	0.6763	0.087*	
H12C	0.5875	0.8406	0.6270	0.087*	
C13	−0.0127 (4)	0.9782 (2)	0.29629 (16)	0.0508 (5)	
H13A	−0.1896	0.9451	0.3122	0.061*	
H13B	0.0210	1.0057	0.2195	0.061*	
H13C	−0.0227	1.0527	0.3210	0.061*	
N1	0.2852 (3)	0.78867 (15)	0.20030 (11)	0.0396 (4)	
H1N	0.108 (5)	0.790 (2)	0.1887 (16)	0.047*	
O1	0.7050 (2)	0.83144 (14)	0.11738 (10)	0.0497 (4)	
O2	0.9467 (3)	0.65764 (18)	−0.11116 (13)	0.0680 (5)	
H2O	1.049 (6)	0.577 (3)	−0.091 (2)	0.082*	
O3	0.7089 (3)	0.57886 (15)	0.04196 (13)	0.0631 (4)	

### Atomic displacement parameters (Å^2^)

**Table:**

	*U*^11^	*U*^22^	*U*^33^	*U*^12^	*U*^13^	*U*^23^
C1	0.0274 (8)	0.0490 (9)	0.0385 (9)	−0.0093 (7)	0.0005 (6)	−0.0158 (7)
C2	0.0415 (9)	0.0474 (9)	0.0448 (9)	−0.0008 (8)	−0.0037 (7)	−0.0159 (8)
C3	0.0538 (12)	0.0562 (11)	0.0483 (11)	0.0068 (9)	−0.0139 (9)	−0.0151 (9)
C4	0.0503 (11)	0.0610 (11)	0.0425 (10)	−0.0052 (9)	−0.0069 (8)	−0.0188 (8)
C5	0.0424 (10)	0.0570 (11)	0.0450 (10)	−0.0039 (8)	0.0014 (8)	−0.0244 (9)
C6	0.0280 (8)	0.0502 (10)	0.0417 (9)	−0.0060 (7)	0.0020 (6)	−0.0159 (7)
C7	0.0226 (8)	0.0466 (9)	0.0426 (9)	−0.0003 (6)	−0.0025 (6)	−0.0190 (7)
C8	0.0281 (9)	0.0769 (13)	0.0448 (10)	0.0025 (9)	−0.0051 (7)	−0.0273 (9)
C9	0.0417 (10)	0.0776 (14)	0.0417 (10)	0.0032 (9)	−0.0022 (8)	−0.0241 (9)
C10	0.0373 (9)	0.0713 (12)	0.0450 (10)	−0.0081 (8)	0.0019 (7)	−0.0332 (10)
C11	0.0688 (14)	0.0530 (11)	0.0553 (12)	0.0092 (10)	−0.0086 (10)	−0.0202 (9)
C12	0.0836 (17)	0.0828 (16)	0.0519 (12)	0.0025 (13)	−0.0207 (11)	−0.0276 (11)
C13	0.0356 (10)	0.0627 (12)	0.0517 (10)	0.0049 (8)	−0.0025 (8)	−0.0226 (9)
N1	0.0213 (7)	0.0588 (9)	0.0420 (8)	−0.0074 (6)	−0.0022 (6)	−0.0206 (7)
O1	0.0221 (6)	0.0789 (9)	0.0516 (7)	−0.0109 (6)	−0.0004 (5)	−0.0249 (7)
O2	0.0600 (10)	0.0758 (10)	0.0631 (9)	0.0032 (8)	0.0198 (7)	−0.0280 (8)
O3	0.0569 (9)	0.0678 (9)	0.0624 (9)	−0.0033 (7)	0.0161 (7)	−0.0256 (8)

### Geometric parameters (Å, °)

**Table:**

C1—C6	1.393 (2)	C8—H8B	1.02 (2)
C1—C2	1.395 (2)	C9—C10	1.491 (3)
C1—N1	1.426 (2)	C9—H9A	0.99 (3)
C2—C3	1.387 (3)	C9—H9B	0.94 (3)
C2—C11	1.502 (3)	C10—O3	1.215 (2)
C3—C4	1.378 (3)	C10—O2	1.311 (2)
C3—H3	0.96 (2)	C11—H11A	0.9600
C4—C5	1.385 (3)	C11—H11B	0.9600
C4—C12	1.507 (3)	C11—H11C	0.9600
C5—C6	1.387 (2)	C12—H12A	0.9600
C5—H5	0.94 (2)	C12—H12B	0.9600
C6—C13	1.506 (2)	C12—H12C	0.9600
C7—O1	1.228 (2)	C13—H13A	0.9600
C7—N1	1.341 (2)	C13—H13B	0.9600
C7—C8	1.509 (2)	C13—H13C	0.9600
C8—C9	1.517 (3)	N1—H1N	0.86 (2)
C8—H8A	1.01 (2)	O2—H2O	0.88 (3)
			
C6—C1—C2	121.06 (15)	C8—C9—H9A	110.4 (14)
C6—C1—N1	119.24 (15)	C10—C9—H9B	107.1 (14)
C2—C1—N1	119.69 (15)	C8—C9—H9B	112.8 (15)
C3—C2—C1	117.88 (17)	H9A—C9—H9B	106 (2)
C3—C2—C11	119.76 (17)	O3—C10—O2	123.15 (19)
C1—C2—C11	122.34 (17)	O3—C10—C9	123.67 (17)
C4—C3—C2	122.86 (18)	O2—C10—C9	113.17 (18)
C4—C3—H3	119.0 (14)	C2—C11—H11A	109.5
C2—C3—H3	118.1 (14)	C2—C11—H11B	109.5
C3—C4—C5	117.53 (17)	H11A—C11—H11B	109.5
C3—C4—C12	121.54 (19)	C2—C11—H11C	109.5
C5—C4—C12	120.93 (19)	H11A—C11—H11C	109.5
C4—C5—C6	122.27 (17)	H11B—C11—H11C	109.5
C4—C5—H5	118.5 (14)	C4—C12—H12A	109.5
C6—C5—H5	119.2 (14)	C4—C12—H12B	109.5
C5—C6—C1	118.35 (16)	H12A—C12—H12B	109.5
C5—C6—C13	120.18 (16)	C4—C12—H12C	109.5
C1—C6—C13	121.46 (15)	H12A—C12—H12C	109.5
O1—C7—N1	123.22 (15)	H12B—C12—H12C	109.5
O1—C7—C8	121.66 (15)	C6—C13—H13A	109.5
N1—C7—C8	115.11 (14)	C6—C13—H13B	109.5
C7—C8—C9	112.74 (15)	H13A—C13—H13B	109.5
C7—C8—H8A	106.9 (13)	C6—C13—H13C	109.5
C9—C8—H8A	107.8 (13)	H13A—C13—H13C	109.5
C7—C8—H8B	105.4 (13)	H13B—C13—H13C	109.5
C9—C8—H8B	112.3 (13)	C7—N1—C1	123.64 (14)
H8A—C8—H8B	111.7 (18)	C7—N1—H1N	117.1 (14)
C10—C9—C8	114.18 (18)	C1—N1—H1N	118.7 (14)
C10—C9—H9A	106.1 (14)	C10—O2—H2O	111.1 (18)
			
C6—C1—C2—C3	−2.6 (3)	N1—C1—C6—C5	−179.01 (15)
N1—C1—C2—C3	178.60 (16)	C2—C1—C6—C13	−177.02 (16)
C6—C1—C2—C11	175.73 (18)	N1—C1—C6—C13	1.8 (2)
N1—C1—C2—C11	−3.1 (3)	O1—C7—C8—C9	20.7 (3)
C1—C2—C3—C4	1.1 (3)	N1—C7—C8—C9	−160.51 (18)
C11—C2—C3—C4	−177.3 (2)	C7—C8—C9—C10	63.5 (3)
C2—C3—C4—C5	0.9 (3)	C8—C9—C10—O3	4.2 (3)
C2—C3—C4—C12	−179.8 (2)	C8—C9—C10—O2	−174.88 (17)
C3—C4—C5—C6	−1.3 (3)	O1—C7—N1—C1	−1.2 (3)
C12—C4—C5—C6	179.3 (2)	C8—C7—N1—C1	179.97 (16)
C4—C5—C6—C1	−0.2 (3)	C6—C1—N1—C7	116.28 (19)
C4—C5—C6—C13	179.05 (18)	C2—C1—N1—C7	−64.9 (2)
C2—C1—C6—C5	2.2 (3)		

### Hydrogen-bond geometry (Å, °)

**Table:**

*D*—H···*A*	*D*—H	H···*A*	*D*···*A*	*D*—H···*A*
N1—H1N···O1^i^	0.86 (2)	2.10 (2)	2.9368 (18)	163.6 (19)
O2—H2O···O3^ii^	0.88 (3)	1.80 (3)	2.679 (2)	172 (3)

Symmetry codes: (i) *x*−1, *y*, *z*; (ii) −*x*+2, −*y*+1, −*z*.
